# CircRNA-9119 suppresses poly I:C induced inflammation in Leydig and Sertoli cells via TLR3 and RIG-I signal pathways

**DOI:** 10.1186/s10020-019-0094-1

**Published:** 2019-06-13

**Authors:** Le Qin, Jie Lin, Xiaoxiao Xie

**Affiliations:** 1 Department of Pediatric Surgery, The Second Affiliated Hospital and Yuying Children’s Hospital of Wenzhou Medical University, Zhejiang, Wenzhou, China; 2 Department of Radiology, The Second Affiliated Hospital and Yuying Children’s Hospital of Wenzhou Medical University, No. 109, West College Road, Lucheng District, Wenzhou, 325027 Zhejiang China

**Keywords:** Orchitis, Inflammation, circRNA-9119, miR-26a, miR-136, TLR3, RIG-I

## Abstract

**Background:**

Circular RNAs (circRNAs) contribute to the epigenetic modulation of pathological and physiological conditions. The understanding of the impact of circRNAs on generation of testicular inflammatory reactions is insufficient.

**Methods:**

Our research adopted a poly I:C-triggered testicular inflammation murine model and cell assays.

**Results:**

Microarray data and quantitative evaluation revealed the elevation in the concentrations of Toll-like receptor 3 (TLR3), circRNA-9119, and retinoic acid inducible gene-I (RIG-I) and repression in the levels of miR-136 and miR-26a. Inhibition of circRNA-9119 expression impaired the inflammatory reactions in the separated Leydig and Sertoli cells subjected to poly I:C treatment. CircRNA-9119 suppressed the expression of miR-136 and miR-26a by acting as a microRNA sponge. miR-136 and miR-26a repressed the expression of RIG-I and TLR3 through the expected target region in Leydig and Sertoli cells in vitro. Inhibition of miR-136 and miR-26a expression, at least in part, restored the expression of inflammatory cytokines, which were inhibited upon circRNA-9119 expression silencing. Furthermore, the expression of circRNA-9119 was positively associated with RIG-I and TLR3 mRNA and protein levels. The expression of inflammatory genes triggered by poly I:C treatment was noticeably suppressed after RIG-I and TLR3 knockout.

**Conclusions:**

Our results suggest that circRNA-9119 may serve as a competing endogenous RNA that insulated miR-136 and miR-26a and consequently defended RIG-I and TLR3 mRNAs against miR-26a/miR-136-mediated inhibition of testicular cells. Moreover, RIG-I and TLR3 contributed to the modulation of poly I:C-triggered inflammatory cytokine generation during orchitis in testicular cells.

**Electronic supplementary material:**

The online version of this article (10.1186/s10020-019-0094-1) contains supplementary material, which is available to authorized users.

## Background

In the process of generation of mammalian sperms, germ cells give rise to numerous innovative antigens after the development of immunocompetence (Yule et al. 1988). In the testis, no damaging immune reactions are stimulated via antigens under physiological circumstances. However, in several pathological circumstances such as trauma, infection, and inflammatory reactions, immunosuppression could be counteracted and may lead to chronic autoimmune orchitis, which may contribute to male infertility (Schuppe et al. 2008). Therefore, the understanding of the etiology of orchitis may assist in the development of innovative strategies to avoid and treat this illness.

Circular RNAs (circRNAs) are regarded as RNAs with loop structures produced via abnormal transcript splicing (Jeck et al. 2013; Rybak-Wolf et al. 2015). CircRNAs are thought to perform crucial functions in various biological reactions, including cell migration, proliferation, and differentiation (Chen et al. 2015; Ebbesen et al. 2016). Few studies have proved the impact as well as mechanisms of circRNAs in orchitis. CircRNAs have been demonstrated to contribute to inflammatory reactions in other cells or tissues. For instance, circRNA-ITCH is displayed in sense orientation to the known protein-coding gene *ITCH*. *ITCH* was recognized when *ITCH* disturbance triggered a lethal autoimmune inflammation (Perry et al. 1998). Ng et al. revealed that the circRNA RasGEF1B acts as a positive modulator of intercellular adhesion molecule 1 (ICAM-1) of the Toll-like receptor 4 (TLR4)/lipopolysaccharide (LPS) pathway, an essential signaling pathway in inflammatory reactions (Ng et al. 2016). The majority of assays have been conducted using cells in steady conditions, which may not highlight the expression of circRNAs in various types of inflammatory reactions (Perry et al. 1998) (Ng et al. 2016). One of these include the transcriptomic reaction of testicular cells after exposure to inflammation triggers. MicroRNAs (miRNAs) modulate gene expression via binding to the 3′-untranslated region (UTR) of the complementary nucleotides of their target mRNAs (Fujii et al. 2018; Hayes et al. 2014). CircRNAs may serve as miRNA sponges and regulate their transcription during various disease development (Rong et al. 2017). Furthermore, miRNAs serve as crucial contributors of orchitis. For instance, it was previously revealed that multiple inflammation-associated miRNAs were inhibited in response to LPS exposure in murine testis models (Parker and Palladino 2017). Moreover, let-7, miR-17, and miR-449a exerted overlapping regulatory effects on male fertility. These miRNAs modulate male germ cell generation and self-renewal (miR-34a/c, let-7, and miR-200c), avoid germ cell death (miR-34c), and target multiple steps of spermatogenesis (miR-34a/c) (Bouhallier et al. 2010; Yao et al. 2015). As a consequence, it is suggested that infections and immune stimulation in testes may induce inflammatory reactions through the modulation of miRNA levels that may be crucial for the normal functioning of testes.

To investigate whether circRNA and miRNA exert crucial role in inflammation and functional development of testes, here we evaluate circRNA expression profiles between non-treated testicular cells (Leydig and Sertoli cells) and testicular cells after treatment with poly I:C, which simulates products of RNA virus replication (Li et al. 2005). Moreover, the association between circRNA and some essential inflammatory sensors, including TLR3 and retinoic acid inducible gene-I (RIG-I), was explored.

## Methods

### Animals

C57BL/6 mice were acquired from Laboratory Animal Center of Peking Union Medical College (Beijing, China). RIG-I and TLR3 (RIG-I^−/−^ and TLR3^−/−^) mice were obtained from Jackson Laboratories (Bar Harbor, ME, USA). Mice were kept in pathogen-free conditions of 12 h light/dark cycle with humidity and temperature in control and food and water ad libitum. All procedures associated with mice were in conformity to guidelines of Care and Use of Laboratory Animals approved via Chinese Council on Animal Care.

### Injection of poly I:C into the testis

Poly I:C was locally injected into the testis of mice following the previous report (Zhu et al. 2013). Briefly, pentobarbital sodium (50 mg/kg) was used to anesthetize mice. Testes were removed via operations. One testis was injected with a mixture of 1 μL of lipofectamine RNAiMAX, 0.3 μg of poly I:C, and 10 μL of phosphate-buffered saline (PBS). Testes were rebuilt 6 h subsequent to the injection of poly I:C to evaluate the mRNA and protein expression of inflammatory markers. Every procedure was approved by the Animal Care and Use Committee of the The Second Affiliated Hospital and Yuying Children’s Hospital of Wenzhou Medical University and was in conformity with the guidelines of National Institute of Health.

### Cell separation

Male germ cells were separated from mice aged 5 weeks as mentioned before (Shang et al. 2011; Zhu et al. 2013). Briefly, testes of three mice were separated from capsules and incubated for 15 min with 0.5 mg/mL of collagenase type 1 (Sigma) in DMEM at room temperature under gentle shaking condition. An 80-μm copper mesh was used to filter the suspension and to isolate interstitial cells from seminiferous tubules. Leydig cells were treated with 0.125% trypsin for 5 min to allow detachment after 24 h of incubation. Detachment protocol was not applied to testicular macrophages. Macrophages in Leydig cell preparation were less than 5%, as per F4/80 immunostaining specific for macrophages. Nonadherent cells were obtained to culture germ cells. Purity of germ cells was over 95%, as evident from the morphology of nucleus in 4′,6-diamidino-2-phenylindole (DAPI) staining (Scarpino et al. 1998). Leydig cell purity was > 92% based on staining for 3β-hydroxysteroid dehydrogenase, a marker of Leydig cells (Klinefelter et al. 1987). The germ cell purity was > 95% based on cell nuclear morphology after staining with DDX4. The purity of Sertoli cells was > 95% based on the immunostaining for Wilms tumor nuclear protein 1, a marker of Sertoli cells (Sharpe et al. 2003).

## Transfection

For poly I:C treatment, Leydig and Sertoli cells (5 × 10^5^ cells/well) were seeded in 60-mm or 6-well plates and cultured for 24 h. Cells were subjected to serum starvation for 2 h prior to transfection with 2 μg/mL of poly I:C as instructed. Freshly separated germ cells were transfected with 2 μg/mL poly I:C, using Lipofectamine 2000 reagent (Invitrogen), under optimized conditions (Manna et al. 2009).

For gene silencing using small-interfering RNA (siRNA), six-well plates were seeded with Leydig and Sertoli cells cells (2 × 10^5^ cells/well) and the cells were transfected with 50 nM of siRNA using Lipofectamine 2000 reagent (Invitrogen), under optimized conditions (Manna et al. 2009) and incubated for 24 h.

### Microarray and quantitative assessment

Separated testicular tissues were shock-frozen at once with the help of liquid nitrogen. The specimens (three poly I:C-supplemented and three non-supplemented) were homogenized with TRIzol reagent (Invitrogen). NanoDrop ND-1000 was used to quantify total RNA in every specimen.

Total RNA in every specimen was amplified and transcribed to fluorescent cRNA using random primers, as per Arraystar Super RNA Labeling protocol (Arraystar Inc.). Arraystar Human circRNA Array was used to hybridized the labeled cRNAs. Agilent G2505C Scanner was used to scan arrays after washing the slides.

### Real-time quantitative reverse-transcription polymerase chain reaction (RT-PCR)

Trizol reagent was used for total RNA isolation. DNase 1 without RNase (Invitrogen) was added to the RNA solution to remove any DNA contaminant, and the product was verified with glyceraldehyde-3-phosphate dehydrogenase (GAPDH) PCR amplification and two micrograms of RNA was reverse transcribed. Power SYBR® Green PCR master mix kit (Applied Biosystems, CA, USA) was used to perform PCR on ABI PRISM 7300 real-time cycler (Applied Biosystems). Relative expression levels of transcripts were quantified according to comparative 2^−ΔΔCT^ method mentioned in Applied Biosystems User Bulletin No. 2 (P/N 4303859) (Livak and Schmittgen 2001).

### Western blot analysis

The proteins (15 μg/well) were separated on 10% sodium dodecyl sulfate polyacrylamide gel electrophoresis gel and the separated bands were electronically transferred onto PVDF membranes. Membranes were blocked for 1 h with Tris-buffered saline containing 5% skim milk at room temperature, then was incubated with primary antibodies at 4 °C. Primary antibodies includes: anti-IRF3 antibody (1:1000, SAB4501564, Sigma), anti-phospho-IRF3 antibody (1:1000, SAB4504031, Sigma), anti-p65 antibody (1:2000, ABE347, Sigma), anti-phospho-p65 antibody (1:1000, SAB4300009, Sigma), anti-Actin antibody (1:5000, A2066, Sigma), anti-TLR3 antibody (1:2500, sc-32,232, Santa Cruz), and anti-RIG-I antibody (1:2500, SAB1305861, Sigma). TBS including 0.1% Tween-20 was used to wash the membranes twice before incubation with secondary antibodies. Enhanced chemiluminescence detection kit (Zhongshan Biotechnology Co.) was used to observe the Ag/Ab complex. β-Actin served as the loading control.

### Indirect immunofluorescence assay (IFA)

For IFA of cultivated testicular cells, cells were permeabilized with 0.2% Triton X-100 in PBS for 15 min and then fixed with paraformaldehyde at 25 °C for 30 min. Cells were blocked with 5% normal goat serum and incubated with primary antibodies. Cells were washed twice with PBS and incubated with appropriate secondary antibodies conjugated to fluorescein isothiocyanate (FITC)/tetramethylrhodamine (TRITC) (Zhongshan Biotechnology Co.) for 60 min. Neutral balsam was used to mount cells and cells were visualized under a fluorescence microscope BX-51 (Olympus, Tokyo, Japan).

### Dual-luciferase reporter assay (DLRA)

Luciferase reporter assay was performed to verify miR-136 and miR-26a target genes. The wild-type (WT) and mutant 3′-UTR of TLR3, RIG-I, and circRNA-9119 were adopted. Calibration of luminescence (Luc) was carried out based on firefly luciferase sequence. *Renilla* luciferase was used as a reporter. Cells were transfected with miR mimic or NC and luminescence vectors and incubated for 36 h.

### Histopathology

The severity of inflammation was analyzed in PBS-injected and Poly I:C-injected testis. Testis were processed routinely, embedded in paraffin wax and 3–5 μm thick serial sections were prepared. The first slide was stained with hematoxylin and eosin and the subsequent section was used for immunohistochemistry. Two veterinary anatomic pathologists independently and blindly scored the histologic lesion severity as previously described (Clancy et al. 2018).

### Statistical analysis

Data are expressed as mean ± standard deviation (SD). Differences among groups were evaluated with one-way analysis of variance (ANOVA) and two-tailed Student’s *t*-test. A value of *P* < 0.05 was considered significant.

## Results

### Poly I:C triggered the expression of inflammatory cytokines in mouse testes and separated testicular cells

**Fig. 1:**
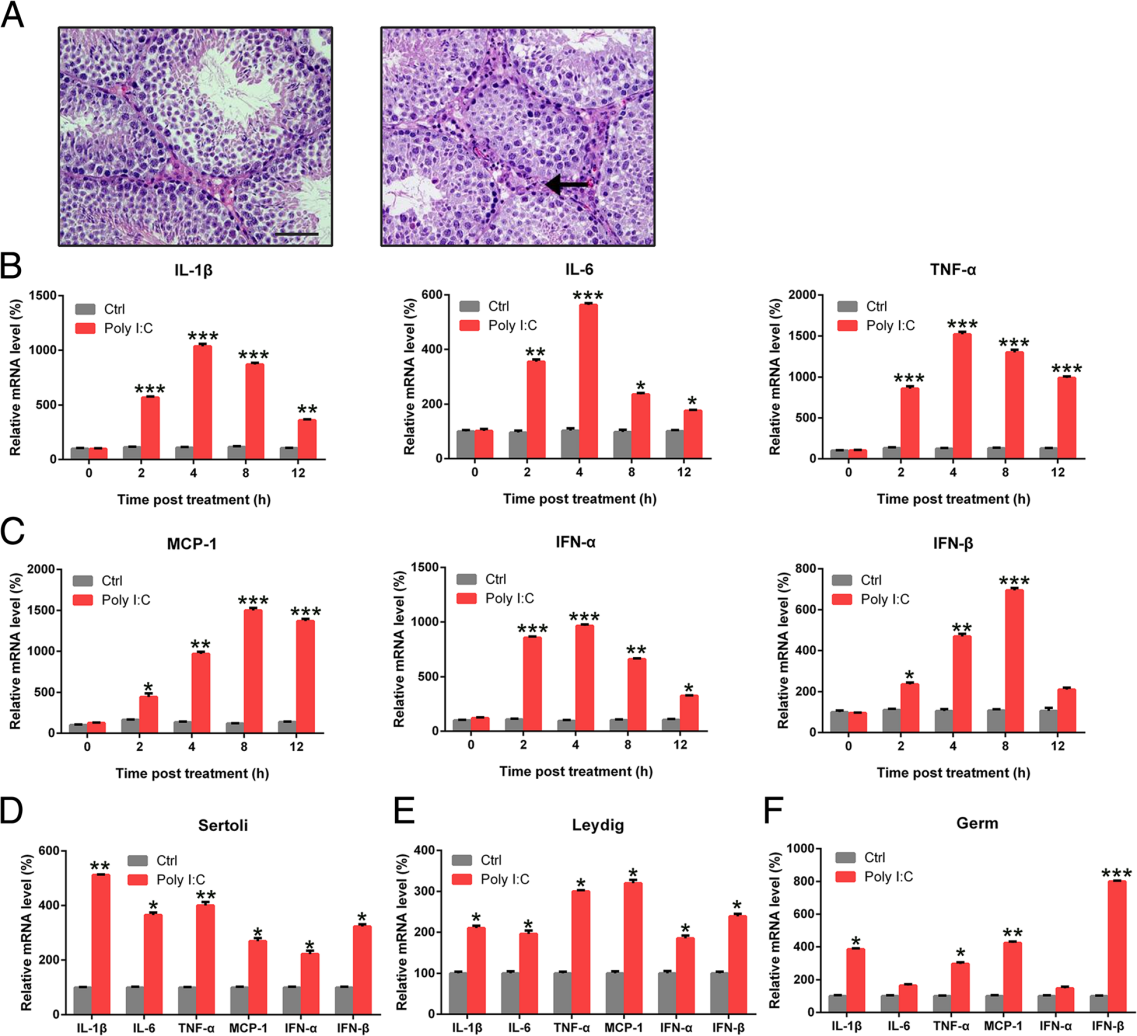
Poly I:C triggered the expression of cytokines. **a** Normal testicle from a sham-infected male. The interstitium contain Leydig (interstitial) cells and blood vessels. Testis from Poly I:C treatment showed mild orchitis. Mild neutrophilic infiltration (arrow) is observed in the interstitium. **b** and **c** Pentobarbital sodium (50 mg/kg) was used to anesthetize mice. Testes were revealed via operation. Testes in the experimental group were injected with 0.3 μg of poly I:C and those from the control group were injected with PBS. At various time points, testes were homogenized and evaluated for the expression of IL-6, MCP-1, IFN-β, IL-1β, TNF-α, and IFN-β with qRT-PCR. **d** Sertoli cells, **e** Leydig cells, and **f** male germ cells were treated for 6 h with poly I:C or PBS. Total RNAs were isolated from cells and the mRNA levels of six cytokines were evaluated with qRT-PCR after normalization to *GAPDH* levels. Cells that did not receive poly I:C injection served as controls (Ctrl). Results are expressed as means ± SEM (*n* = 3 replicates each experiment). *P < 0.05, **P < 0.01, ***P < 0.001

To explore the innate immune response of testicular cells following poly I:C injection, we first examined the histological architecture of the testis after the treatment. Mild orchitis and epididymitis were the most severe inflammatory lesions observed in the testicle injected with Poly I:C (Fig. 1a). No testicular inflammation or epithelial cell necrosis was observed in sham-infected males. We then evaluated the expression of interferons (IFNs), chemokines, and inflammatory cytokines in the testes of infected mice as well as in the germ, Sertoli, and Leydig cells. qRT-PCR analysis revealed that the injection of poly I:C increased the expression levels of interleukin (IL)-6, monocyte chemoattractant protein-1 (MCP-1), IFN-β, IL-1β, tumor necrosis factor (TNF)-α, and IFN-α in a time-dependent manner (Fig. 1b, c). Peak levels of inflammatory cytokines were observed after 4 h of injection, while IFN and chemokine levels were maximum at 8 h. We separated germ, Leydig, and Sertoli cells (Additional file 1: Figure S1) and evaluated the expression levels of six genes at 6 h of poly I:C treatment. qRT-PCR results showed that poly I:C treatment remarkably triggered the expression of these genes in Leydig and Sertoli cells (Fig. 1d, e), but not in germ cells, wherein only IFN-β, IL-1β, MCP-1, and TNF-α levels were increased (Fig. 1f).

**Fig. 2:**
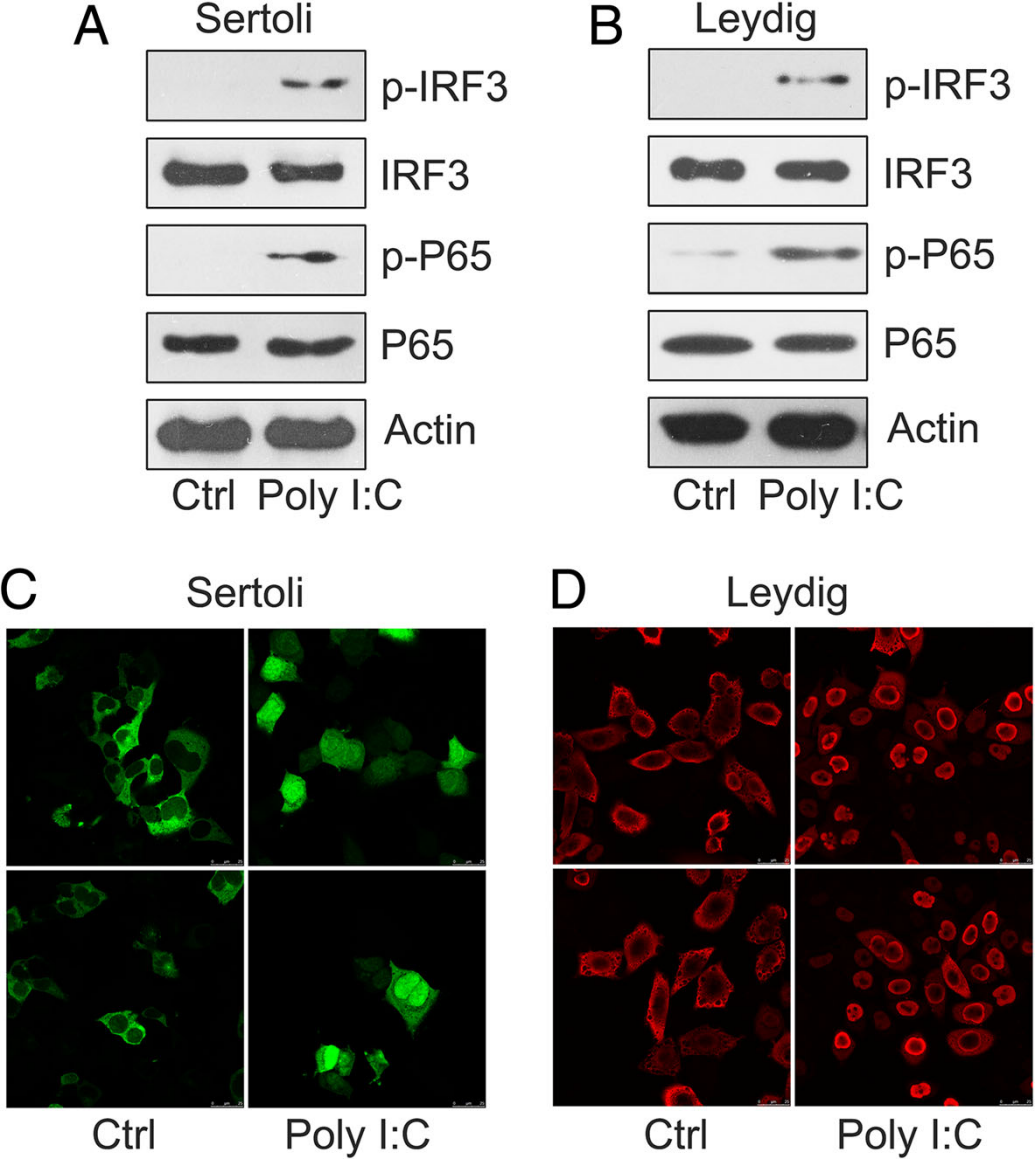
Poly I:C triggered NF-κB and IRF3 expression. **a** Sertoli and **b** Leydig cells were treated for 6 h with 2 μg/mL of poly I:C. Lysates were subjected to western blot analysis and probed with antibodies for phosphorylated IRF3 (p-IRF3) p-P65, total P65, and IRF3. β-Actin served as a loading control. **c** and **d**) IFA was used to study the nuclear translocation of IRF3 and P65. Leydig and Sertoli cells were treated with 2 μg/mL of poly I:C. Distribution of P65 and IRF3 inside the cells was assessed with indirect IFA. Images were obtained for P65 and IRF3 translocation in Leydig (red) and Sertoli (green) cells after 8 h treatment. Cells with no transfection served as controls (Ctrl). Images were independently obtained for at least three experiments

As the phosphorylation of interferon regulator factor 3 (IRF3) and nuclear factor kappa B (NF-κB) is crucial to trigger the expression of cytokines (Wu and Chen 2014), we investigated the phosphorylation level and cellular sites of these transcription factors (TFs) in Leydig and Sertoli cells after treatment with poly I:C. Western blot analysis results showed that poly I:C triggered the phosphorylation of IRF3 and NF-κB (P65) in both Leydig and Sertoli cells at 6 h (Fig. 2a, b). Stimulated IRF3 and P65 were translocated to the nucleus to upregulate cytokine expression. Efficient IRF3 and P65 translocation was observed with IFA in Leydig and Sertoli cells at 8 h of poly I:C treatment (Fig. 2c, d). However, we failed to observe IRF3 and P65 translocation in the control group treated with PBS. These results suggest that the treatment with poly I:C triggered inflammatory reactions in Leydig and Sertoli cells through the stimulation of IRF3 and NF-κB.

### CircRNA expression profile in the testes stimulated with poly I:C

**Fig. 3:**
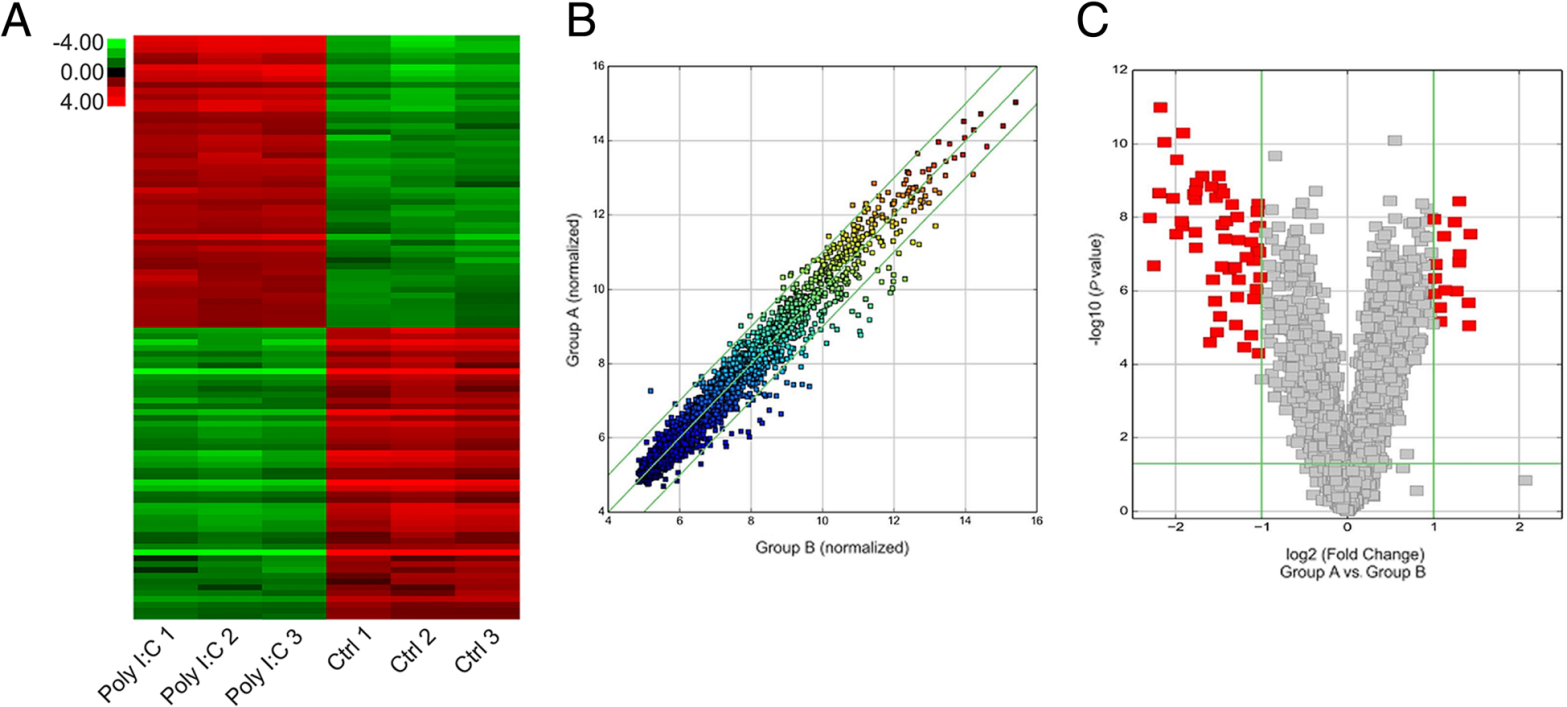
Expression of Various circRNAs in the testes treated with poly I:C. **a** Hierarchical clustering assessment of circRNAs that displayed variation in expression patterns between control and poly I:C treatment groups; every group comprised three individuals (over two-fold difference in expression; *P* < 0.05). Expression values are shown in various colors suggestive of high and low median expression levels. **b** Scatter plot was used to evaluate alterations in circRNA expression between control (group A) and poly I:C treatment (group B) specimens. Values corresponding to X and Y axes in the scatter plot were normalized signal values of specimens (log_2_ scaled). Green lines indicate fold alterations. CircRNAs over the top green line and below the bottom green line indicate over two-fold changes. **c** Volcano plots were built to show fold change values and *P* values. The vertical lines show two-fold upregulation and downregulation between control and poly I:C treatment specimens (A versus B), and the horizontal line shows *P* value. The red point in the plot shows various expression patterns of circRNAs with statistical significance

**Fig. 4:**
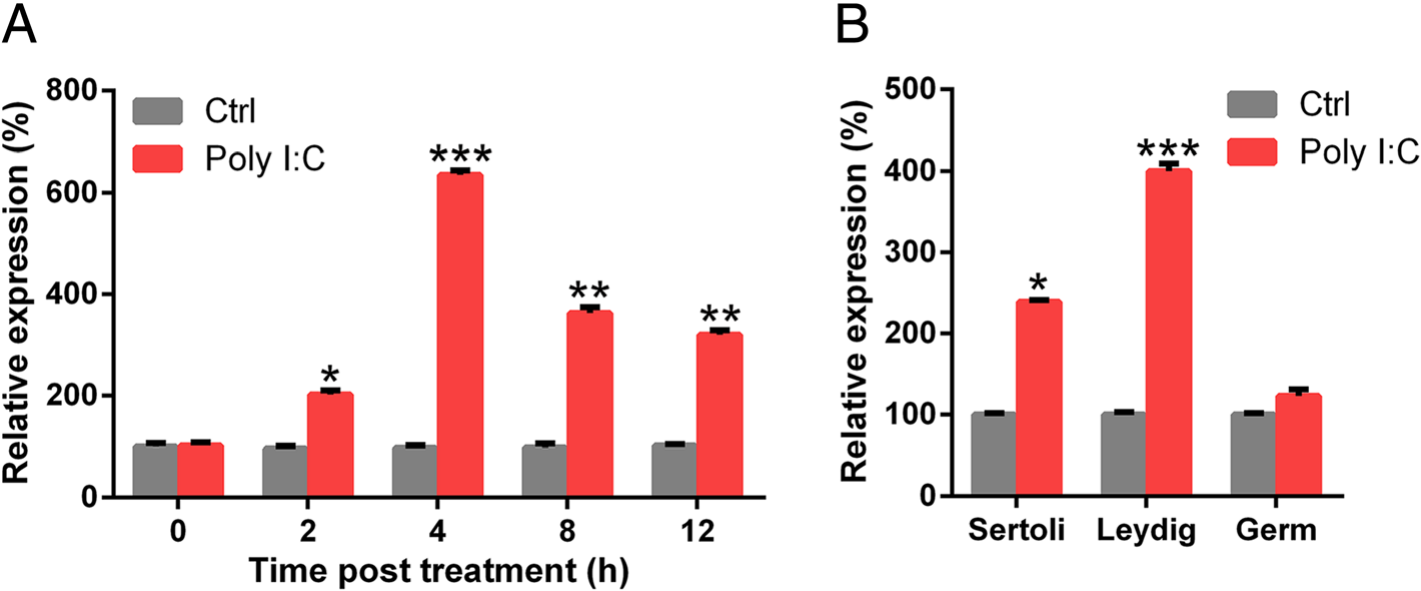
Poly I:C treatment increased the expression of circRNA-9119. **a** Pentobarbital sodium (50 mg/kg) was used to anaesthetize mice. Testes were revealed via operation. Testes in the experimental group received 0.3 μg of poly I:C, while those from the control group were injected with PBS. At various time points, testes were homogenized and examined for circRNA-9119 concentration with qRT-PCR. **b** Sertoli cells, Leydig cells, and male germ cells were separated from C57BL/6 mice after 6 h treatment with poly I:C or PBS. Total RNAs were isolated from cells, while circRNA-9119 concentration was evaluated with qRT-PCR after normalization to *GAPDH* level. Cells without poly I:C treatment served as controls (Ctrl). Results are shown as means ± SEM (*n* = 3 replicates each experiment). *P < 0.05, **P < 0.01, ***P < 0.001

The expression of circRNA was evaluated in poly I:C-treated and control specimens with hierarchical clustering (Fig. 3a). Change in circRNA expression between poly I:C-treated and control specimens was revealed via scatter and volcano plots (Fig. 3b, c). Thirty-five circRNAs showed remarkable expression patterns in Poly I:C-treated specimens as compared with the control specimens. Of these, 25 circRNAs were downregulated and 10 candidates showed upregulated expression in poly I:C-treated specimens. CircRNA-9119 showed the most obvious variation in expression. qRT-PCR analysis showed that the injection of poly I:C resulted in a remarkable elevation in circRNA-9119 concentration in the testes in a time-dependent manner as compared with the control cells (Fig. 4a). Among the poly I:C-treated cells, Leydig and Sertoli cells showed elevated expression of circRNA-9119 as compared with germ cells (Fig. 4b).

### CircRNA-9119 was crucial for inflammatory reactions in testicular cells

**Fig. 5:**
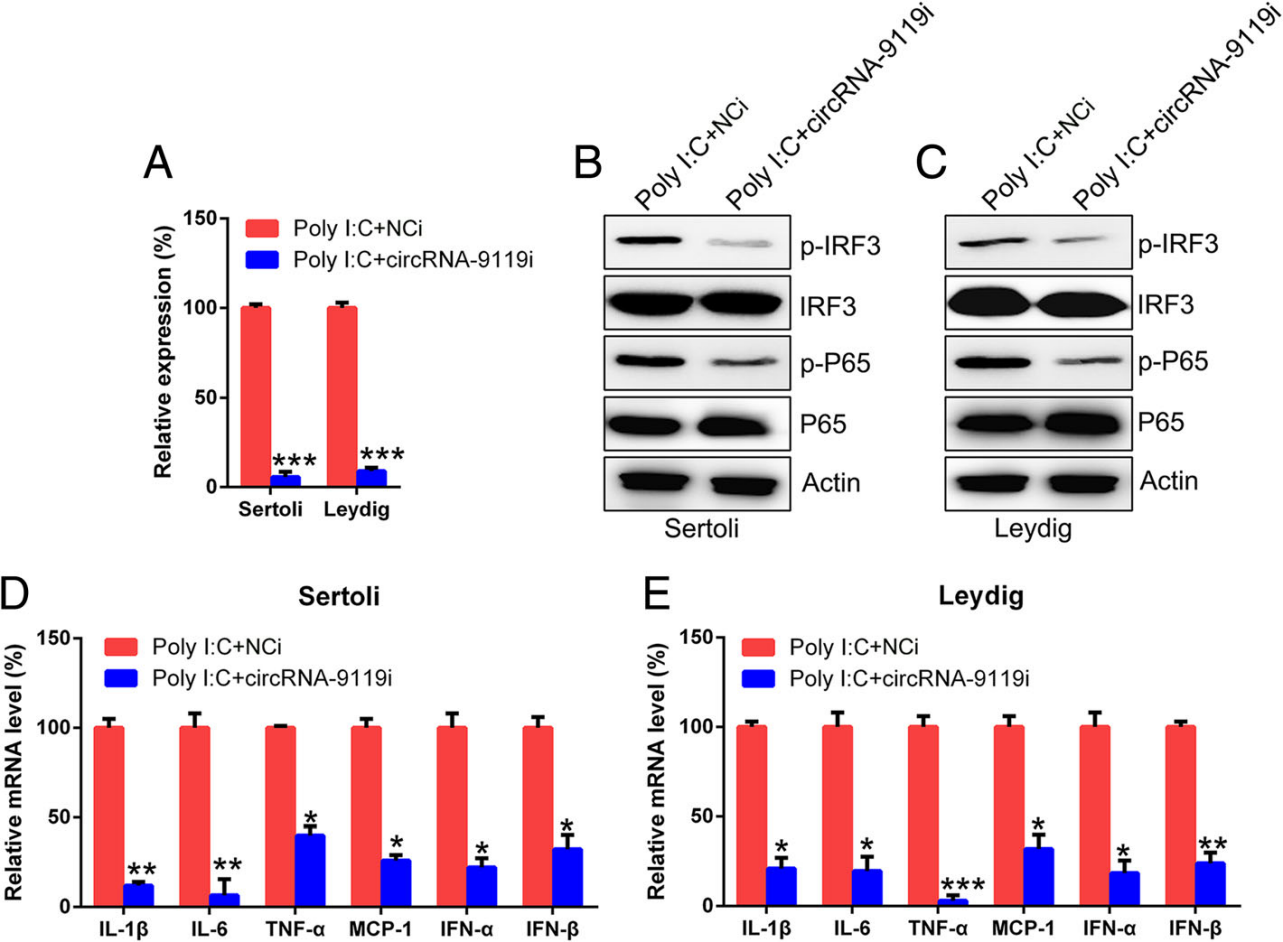
Effect of circRNA-9119 inhibition on the transcription and translation of inflammation-associated genes in testicular cells. **a** Sertoli and Leydig cells were separated from C57BL/6 mice and treated with circRNA-9119 siRNA or NC siRNA prior to treatment with poly I:C or PBS for 6 h. Total RNA and proteins were isolated from cells, and circRNA-9119 level was evaluated with qRT-PCR after normalization to *GAPDH* level. **b** and **c** Western blotting was used to investigate the stimulation of P65 and IRF3 expression and to evaluate the expression of total P65 and IRF3. **d** and **e** qRT-PCR was used to detect the mRNA levels of cytokines after 6 h treatment with poly I:C. Results are expressed as means ± SEM (*n* = 3 replicates each experiment). *P < 0.05, **P < 0.01, ***P < 0.001

To evaluate the contribution of circRNA-9119 to inflammatory reactions in both Leydig and Sertoli cells, circRNA-9119 expression was inhibited in these poly I:C-treated cells. The expression of circRNA-9119 was suppressed in the cells treated with circRNA-9119 siRNA as compared with those treated with NC siRNA (Fig. 5a). We assessed the expression of IRF3 and P65 in Leydig and Sertoli cells after various treatments. Phosphorylation of IRF3 and P65 in these two cells was noticeably inhibited after transfection with circRNA-9119 siRNA, as observed with western blotting (Fig. 5b, c). Furthermore, qRT-PCR was carried out to evaluate the expression of mRNAs of six cytokines after treatment with poly I:C and/or circRNA-9119 siRNA. We observed that the expression of IFNs, chemokines, and inflammatory cytokines reduced after circRNA-9119 expression inhibition (Fig. 5d, e), suggestive of the essential role of circRNA-9119 in inflammatory reactions in testicular cells.

### CircRNA-9119 modulates miR-26a and miR-136 expression

**Fig. 6:**
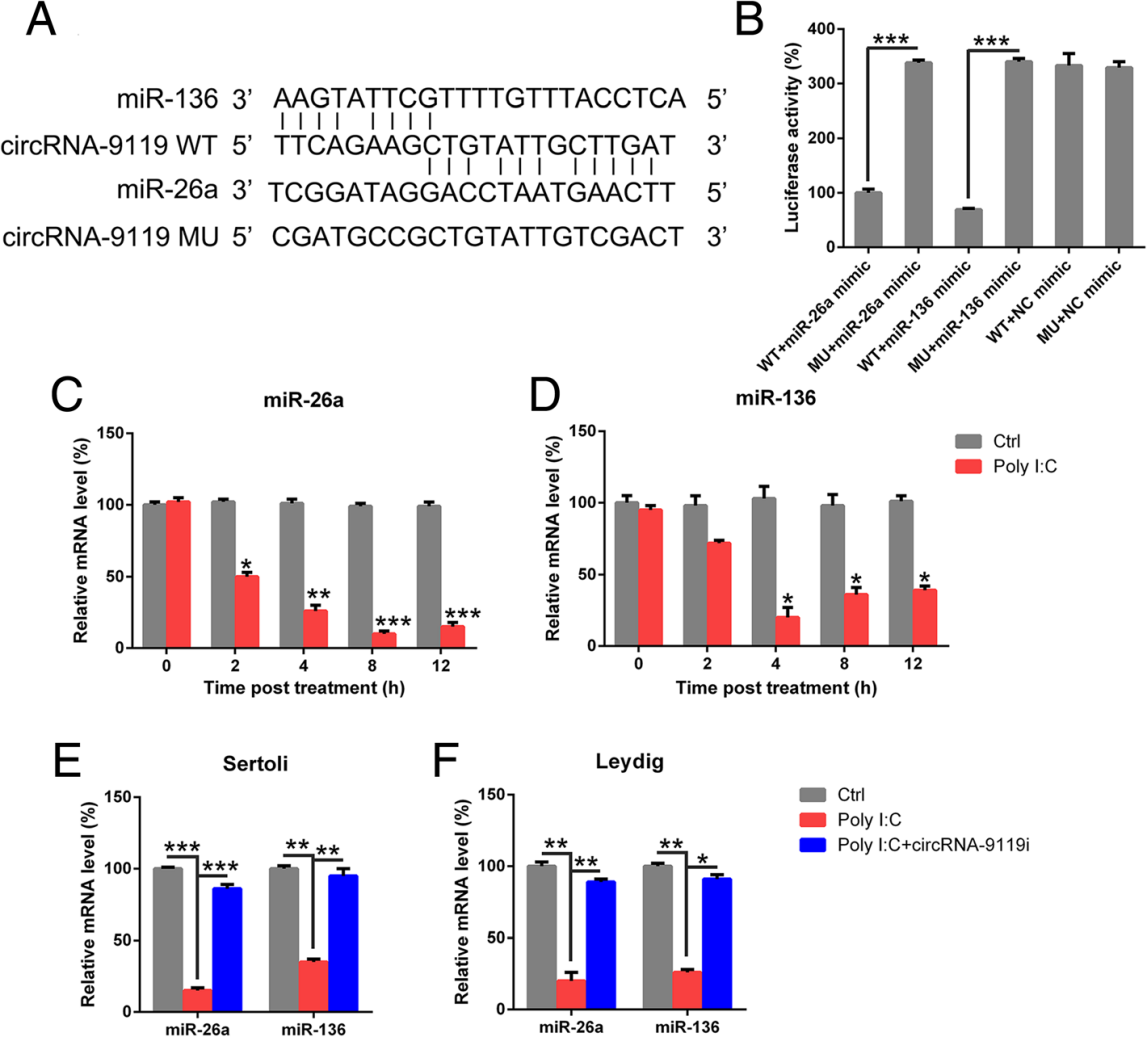
circRNA-9119 targeted miR-26a and miR-136. **a** Graphical illustration of the conserved circRNA-9119-binding motifs in miR-26a and miR-136. **b** Luciferase function was evaluated with luciferase reporter products comprising either WT or mutated (MU) copy of human circRNA-9119 after transfection with miR-26a/miR-136 mimic. Luciferase function was normalized to β-galactosidase level. **c** and **d** Testes in experimental group received injection of 0.3-μg poly I:C, while the control group was injected with PBS. At various time points, testes were homogenized and examined for miR-26a and miR-136 expression by qRT-PCR. **e** and **f** Sertoli and Leydig cells were separated from C57BL/6 mice and transfected with circRNA-9119 siRNA or NC siRNA prior to treatment with poly I:C or PBS for 6 h. Total RNAs were isolated from cells, and miR-26a and miR-136 concentrations were evaluated with qRT-PCR after normalization to *GAPDH* level. Results are shown as means ± SEM (*n* = 3 replicates each experiment). *P < 0.05, **P < 0.01, ***P < 0.001

Emerging evidences have suggested that circRNAs modulate miRNA target genes by acting as miRNA sponges. circRNA-9119 targets miR-26a in the endometrium (Zhang et al. 2018) (Fig. 6a). We hypothesized that circRNA-9119 targets miR-26a and mediates the downstream activities during the inflammation of testicular cells. Bioinformatic assessment was carried out to predict the targets of circRNA-9119. We found that circRNA-9119 targets miR-136 (Fig. 6a). We performed DLRA to explore the direct association between circRNA-9119 and miR-26a/miR-136 (Fig. 4b). Luciferase function was inhibited by 70 and 80% in HEK293T cells transfected with miR-26a mimic and miR-136 mimic fused to the WT circRNA-9119, respectively, as compared with the control cells (Fig. 6b). We assessed the expression of miR-26a and miR-136 in testicular specimens from mice treated or untreated with poly I:C injection. The expression of miR-26a and miR-136 was downregulated in poly I:C-treated mice as compared with the control mice (Fig. 6c, d). Furthermore, the levels of miR-26a and miR-136 were evaluated in separated Leydig and Sertoli cells treated with circRNA-9119 siRNA to explore the association between miRNA and circRNA-9119. We found that poly I:C treatment downregulated the expression of miR-26a and miR-136 in both cells and the inhibition of circRNA-9119 expression remarkably restored miR-26a and miR-136 expression (Fig. 6e, f). Thus, circRNA-9119 reduced the expression level of miR-136 and miR-26a in testicular cells.

### Inflammatory activity of circRNA-9119 is associated with miR-136 and miR-26a

**Fig. 7:**
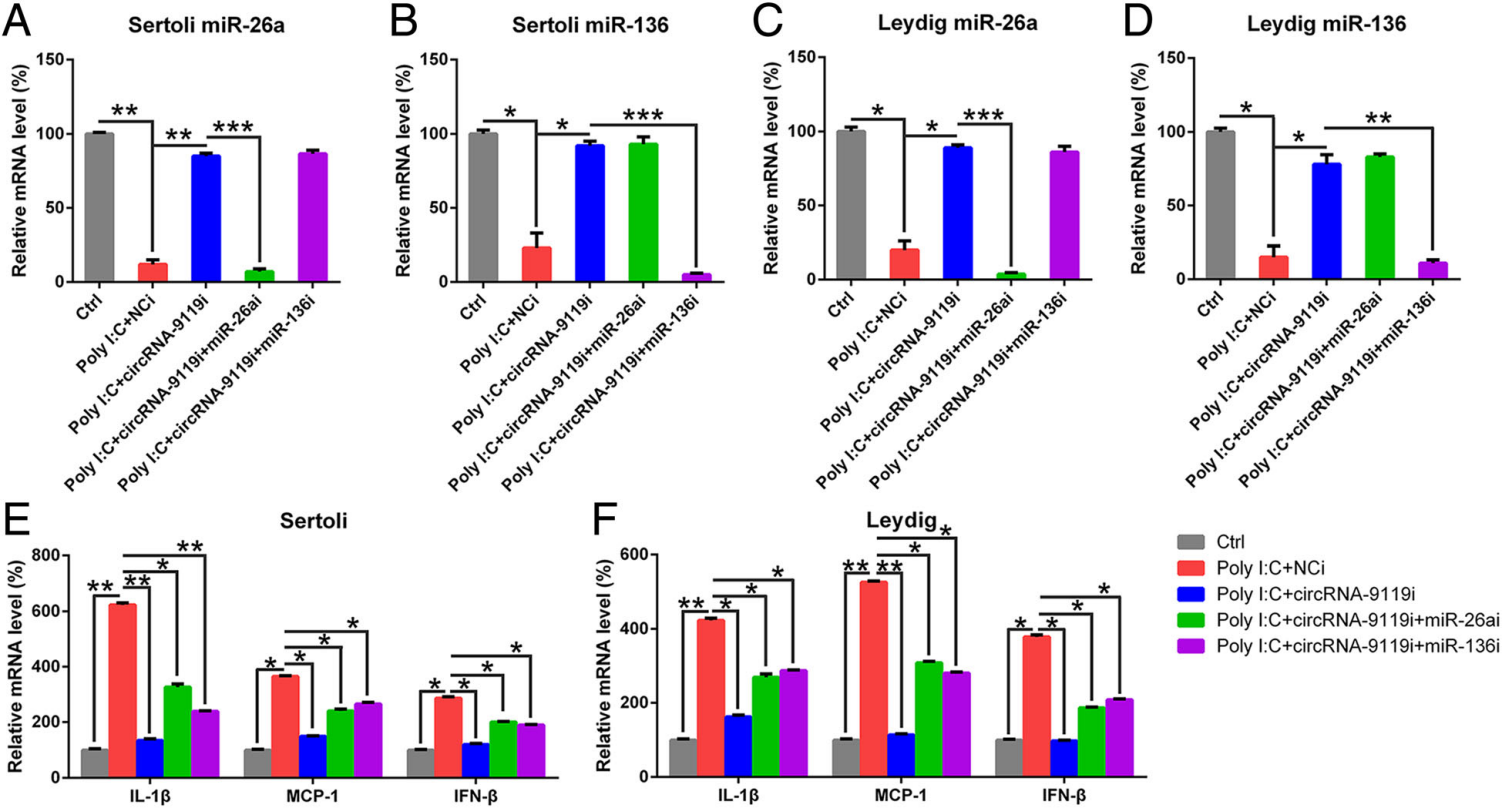
miR-26a and miR-136 contributed to circRNA-9119-mediated inflammatory reactions. **a**-**d** Sertoli and Leydig cells were separated from C57BL/6 mice and co-transfected with circRNA-9119 siRNA and/or miR-26a/miR-136/NC siRNA, prior to treatment with poly I:C or PBS for 6 h. Total RNA was isolated from cells and miR-26a and miR-136 levels were evaluated with qRT-PCR after normalization to *GAPDH* level. **e** and **f** qRT-PCR was used to evaluate the expression of IL-1β, MCP-1, and IFN-β in Sertoli and Leydig cells after 6 h of poly I:C treatment. Results are expressed as means ± SEM (*n* = 3 replicates each experiment). *P < 0.05, **P < 0.01, ***P < 0.001

Separated Leydig and Sertoli cells were co-transfected with circRNA-9119 siRNA and/or miR-26a/miR-136 siRNA to evaluate the impact of miR-26a and mR-136 on circRNA-9119 activities. qRT-PCR revealed the downregulation in miR-136 and miR-26a expression in the two cell types after siRNA treatment (Fig. 7a and d). Transfection of cells with miR-26a/miR-136 siRNA noticeably counteracted miRNA upregulation, which was associated with the inhibition of circRNA-9119 expression. Further assays were carried out to measure the levels of inflammatory cytokines (IL-1β), IFN-β, and chemokine (MCP-1) after miR-26a or miR-136 expression downregulation. Treatment of cells with poly I:C induced inflammation in the two cell types, but circRNA-9119 expression inhibition resulted in a decrease in the expression level of IFN-β, IL-1β, and MCP-1 to normal levels. miR-26a or miR-136 downregulation in the cells transfected with circRNA-9119 siRNA resulted in the recovery in the expression of cytokines, at least in part, observed after poly I:C treatment (Fig. 7c, d). Thus, miR-26a as well as miR-136 are accounted for the inflammatory activities of circRNA-9119.

### miR-26a and miR-136 modulates the activities of LPS-triggered chondrocytes by targeting TLR3 and RIG-I, respectively

**Fig. 8:**
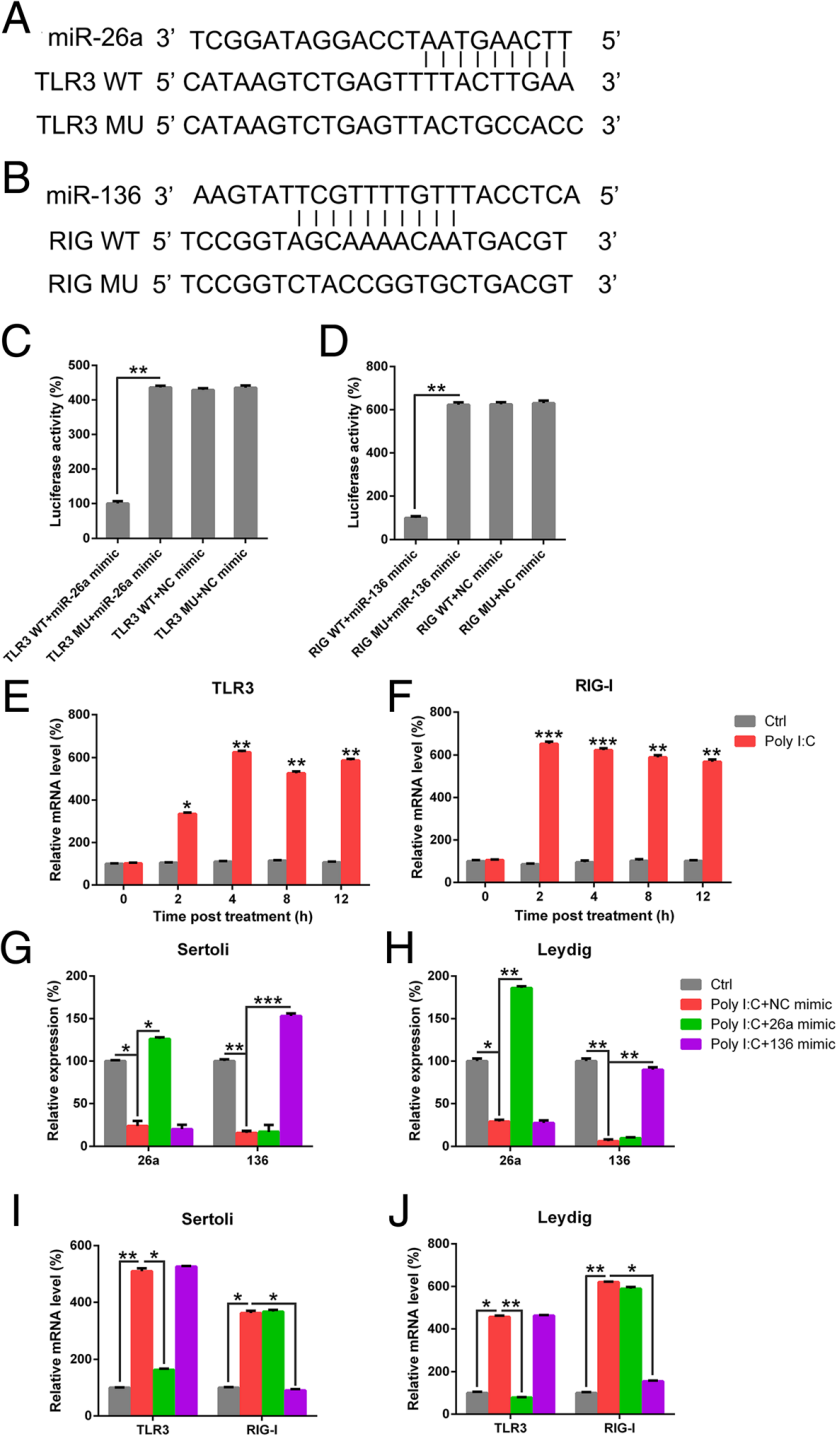
miR-26a and miR-136 target TLR3 and RIG-I, respectively. **a** and **b** Graphical illustrations of the conserved miR-26a- and miR-136-binding motifs at the 3′-UTR of TLR3 and RIG-I, respectively. **c** and **d** Luciferase function was evaluated with luciferase reporter products comprising either WT or mutated (MU) copy of TLR3 and RIG-I after transfection with miR-26a/miR-136 mimic. Luciferase function was normalized to β-galactosidase level. **e** and **f** Testes in the experimental group received 0.3 μg of poly I:C, while those form the control group were injected with PBS. At various time points, testes were homogenized and examined for mRNA levels of *TLR3* and *RIG-I* with qRT-PCR. **g**-**j** Sertoli and Leydig cells were separated from C57BL/6 mice and transfected with miR-26a mimic, miR-136 mimic, or NC mimic prior to treatment with poly I:C or PBS for 6 h. Total RNA was isolated from cells, and miR-26a, TLR3, RIG-I, and miR-136 levels were evaluated with qRT-PCR after normalization to *GAPDH* level. Results are expressed as means ± SEM (*n* = 3 replicates each experiment). *P < 0.05, **P < 0.01, ***P < 0.001

miR-26a exhibits binding sites at the 3′-UTR of TLR3, while miR-136 binds to the 3′-UTR of RIG-I (Fig. 8a, b), consistent with the results of bioinformatic analyses in previous studies (Jiang et al. 2014a; Zhao et al. 2015). DLRA results confirmed that miR-26a and miR-136 bind to TLR3 and RIG-I, respectively. Transfection of cells with miR-136 or miR-26a mimic resulted in the inhibition of luciferase function, owing to the binding to TLR3 or RIG-I, respectively (Fig. 8c, d). Moreover, RIG-I and TLR3 expression increased in the testes treated with poly I:C as compared with the control testes after 2 and 4 h of treatment, respectively (Fig. 8e, f). To verify the association between miR-26a/miR-136 and the expression of TLR3/RIG-I, miR-26a or miR-136 mimic was used to increase the expression of miR-26a or miR-136, respectively, in Leydig and Sertoli cells triggered with poly I:C. qRT-PCR results revealed that the expression of the two miRNAs increased in Leydig and Sertoli cells (Fig. 8g, h). The upregulation in miR-26a or miR-136 expression resulted in an obvious suppression in the mRNA levels of *TLR3* or *RIG-I*, respectively (Fig. 8i, j). Thus, miR-26a and miR-136 targeted the 3′-UTR of TLR3 and RIG-I, respectively, consequently leading to the downregulation of protein expression.

### Both TLR3 and RIG-I modulate circRNA-9119-mediated inflammatory reactions in testicular cells

**Fig. 9:**
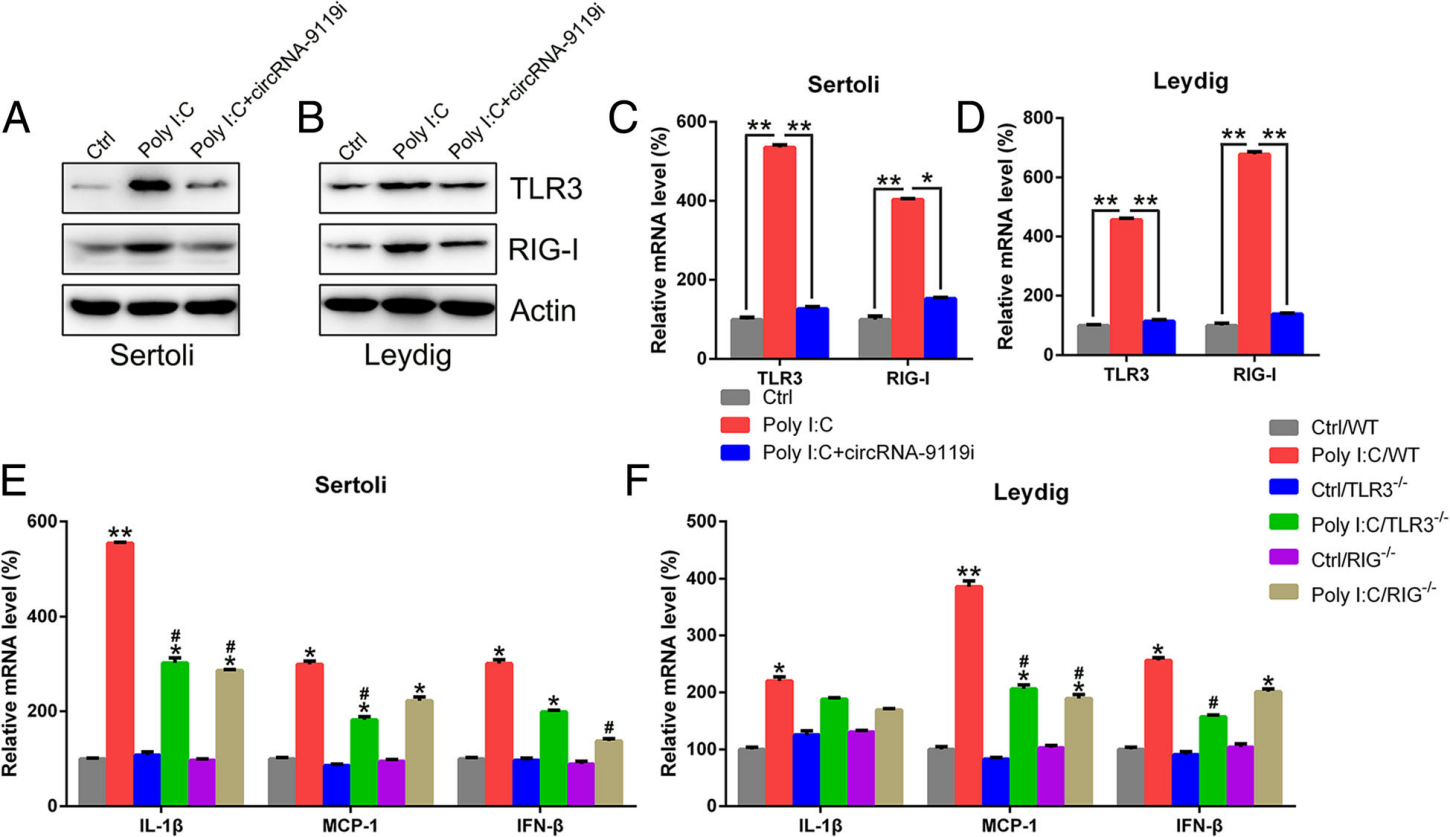
CircRNA-9119-modulated testicular inflammatory reactions were in part attributed to TLR3 and RIG-I expression. Sertoli and Leydig cells were separated from C57BL/6 mice and transfected with circRNA-9119 siRNA or NC siRNA prior to treatment with poly I:C or PBS for 6 h. Total RNA and proteins were isolated from cells. Translation **a** and **b** and transcription **c** and **d** of TLR3 and RIG-I were investigated with western blotting and qRT-PCR, respectively. **e** and **f** Sertoli and Leydig cells were separated form WT C57BL/6, TLR3^−/−^, and RIG-I^−/−^ mice before treatment with poly I:C or PBS (Ctrl) for 6 h. qRT-PCR was performed to examine the expression level of IL-1β, IFN-β, and MCP-1 in Leydig and Sertoli cells. Results are expressed as means ± SEM (*n* = 3 replicates each experiment). *P < 0.05, **P < 0.01 versus respective Ctrl group; ^#^*P* < 0.05 versus poly I:C/WT group

miR-26a and miR-136 were thought to participate in the circRNA-9119-mediated inflammatory reactions by targeting TLR3 and RIG-I, respectively, which were commonly recognized as crucial sensors for testicular inflammation-associated pathways (Zhao et al. 2014). To evaluate the direct impact of RIG-I and TLR3 on circRNA-9119-mediated inflammatory reactions in Leydig and Sertoli cells stimulated with poly I:C, we inhibited the expression of circRNA-9119 in testicle cells treated with poly I:C. Western blotting and qRT-PCR results showed that TLR3 and RIG-I expression was inhibited in the cells lacking circRNA-9119 expression (Fig. 9a and d), indicating that circRNA-9119 expression was positively related to TLR3/RIG-I. Moreover, TLR3 and RIG-I knockout mice were used to evaluate the impact of the absence of TLR3 and RIG-I on inflammatory testicular cells after poly I:C stimulation. Poly I:C treatment noticeably upregulated the expression of three cytokines (IL-1β, MCP-1, and IFN-β) in TLR3 knockout, RIG-I knockout, and WT Sertoli cells. TLR3 knockout cells showed a remarkable suppression in the concentration of MCP-1 and IL-1β, and RIG-I knockout cells showed suppressed expression of IFN-β and IL-1β as compared with WT cells (Fig. 9e). In Leydig cells, TLR3 knockout reduced the levels of MCP-1 and IFN-β, while RIG-I knockout inhibited MCP-1 expression (Fig. 9f). The above findings indicate that circRNA-9119 exhibited inflammatory activities through the modulation of RIG-I as well as TLR3 expression.

## Discussion

CircRNAs are gene regulators that participate in multiple physiological functions and pathological reactions, and may serve as miRNA sponges (Hansen et al. 2013). Several circRNAs have been recently identified and shown to exert important regulatory effects as miRNA sponges (Rong et al. 2017). However, most studies on circRNAs have focused on tumorigenesis, and no studies have reported the role of circRNAs in testis-related pathologies. In the present study, we found that circRNA-9119 is a regulatory circRNA involved in the inflammation of testes and serves as a sponge for miR-26a and miR-136 in Sertoli and Leydig cells in response to poly I:C stimulation.

Both miR-26a and miR-136 are conserved miRNAs that play an important role in cell differentiation, development, and growth (Zhang et al. 2015; Yang et al. 2012). It has been reported that miR-26a modulates cell proliferation by mediating TLR9 expression (Jiang et al. 2014b) and regulates the survival of various cells by targeting *SODD* gene (Reuland et al. 2013). miR-26a also reversely modulates TLR3 expression in murine macrophages and attenuates pristine-triggered arthritis (Jiang et al. 2014a). miR-136 exerts a regulatory role in non-small cell lung cancer cells by stimulating the extracellular signal-regulated kinase (ERK)1/2 pathway through protein phosphatase 2 regulatory subunit B alpha (PPP2R2A) (Shen et al. 2014). Overexpression of miR-136 results in the inhibition of metastasis-associated traits in lung adenocarcinoma cells via Smad2 and Smad3 (Yang et al. 2014). In addition, miR-136 serves as a modulator of RIG-I-mediated innate immunity, which counteracts influenza A virus H5N1 replication in A549 cells (Zhao et al. 2015). Together, these researches indicate that miR-26a and miR-136 possess important functions in different biological and pathological processes. We confirmed the low abundance of miR-26a and miR-136 and high levels of circRNA-9119 in inflammatory testicular cells. qRT-PCR results further indicated that both miRNAs were downregulated by circRNA-9119. Furthermore, miR-26a and miR-136 expression inhibition reversed the pro-inflammatory effects of circRNA-9119. Thus, we hypothesize that circRNA-9119 may contribute to the modulation of orchitis by regulating the expression levels of miR-26a and miR-136 in testicle cell lines in vitro.

Testes are vulnerable to multiple pathogens arising from blood or those from the genitourinary tract (Zhao et al. 2014). Testicular cells must get over the immune privilege aiming at exerting a proper and efficient local reaction counteracting invading pathogens. This reaction occurs through appropriate antimicrobial innate immune reactions (Zhao et al. 2014). Innate immunity of testicles is extremely crucial during impaired systemic immunity (Zhao et al. 2014). Macrophages are a major population of cells that represent approximately 20% of the total testicular interstitial cells in mice under physiological conditions (Hedger 2002). The macrophages have an important function in regulating the development and steroidogenesis of Leydig cells in rats (Hutson 2006). Macrophages belong to the family of antigen-presenting cells. However, testicular macrophages exhibit relatively low inflammatory responses and high immunosuppressive properties compared with the macrophages located in other tissues (Kern et al. 1995). Therefore, we did not choose macrophages in the present study. But its molecular mechanism on inflammation will be researched in future. Both Sertoli cells and Leydig cells are different functional testicular cell types. But their manifestation during testicular innate immune was reported to be regulated by TLR and RLR similarly (Shang et al. 2011; Riccioli et al. 2006; Yoneyama et al. 2005; Wu et al. 2016). Pattern recognition receptors (PRRs) are a group of receptors that are stimulated via conserved structures of microorganisms, also called as pathogen-associated molecule patterns (PAMPs). TLRs are well investigated and 13 TLRs have been identified in mammals. As a double-stranded RNA sensor in the cytoplasm, RIG-I-like receptors comprise two functional parts, MDA5 and RIG-I. Murine Leydig cells exhibit expression and activities of TLR3 and TLR4, while RIG-I and MDA5 expression is frequent in mouse Leydig cells. MDA5 was also identified in spermatids (Zhu et al. 2013). Poly I:C stimulates innate immune reactions in Sertoli and Leydig cells via TLR3 and RIG-I-mediated pathways (Zhu et al. 2013; Zhang et al. 2013; Palladino et al. 2007). Our research demonstrates that poly I:C injection and transfection drastically upregulated the expression of pro-inflammatory cytokines, chemokines, and IFNs not only in Leydig but also in Sertoli cells. Moreover, stimulation of testicular cells derived from TLR3^−/−^ and RIG^−/−^ mice with poly I:C resulted in the reduction in the expression of inflammatory cytokines as compared with the cells from the WT mice, suggestive of the important contribution of TLR3 and RIG-I in the initiation of testicular immune reaction. We confirmed that the expression miR-26a and miR-136 negatively correlated with the concentrations of TLR3 and RIG-I, which positively correlated with circRNA-9119 level in the inflammatory testicular cells. DLRA results indicated that the 3′-UTR of TLR3 and RIG-I was targeted by miR-26a and miR-136, respectively. Therefore, TLR3 and RIG-I upregulation may contribute to the pro-inflammatory activities of circRNA-9119 in orchitis.

## Conclusions

In conclusion, we demonstrate the pro-inflammatory function of circRNA-9119 in the testes and isolated Sertoli and Leydig cells in response to poly I:C stimulation. circRNA-9119 participates in testicular immune reaction that counteracts mimetic viral infections. circRNA-9119 acted as a sponge for both miR-26a and miR-136, which in turn target TLR3 and RIG-I, respectively, the two essential molecules in orchitis. The present study results provide an evidence of the significance of the inflammation-related circRNA-9119-miR-26a/miR-136-TLR3/RIG-I axis for innate immune reactions of testes.

## Additional file

Additional file 1: **Figure S1.** Immunofluorescence assay of Leydig, Sertoli, and Germ cells. (DOCX 469 kb)

## Data Availability

All data generated or analysed during this study are included in this published article and its supplementary information files.

## Abbreviations

ANOVA: Analysis of variance
circRNAs: circular RNAs
DAPI: Diamidino-2-phenylindole
DLRA: Dual-luciferase reporter assay
ERK: Extracellular signal-regulated kinase
FITC: Fluorescein isothiocyanate
GAPDH: Glyceraldehyde-3-phosphate dehydrogenase
ICAM-1: Intercellular adhesion molecule 1
IFA: Immunofluorescence assay
IFNs: Interferons
IL: Interleukin
IRF3: Interferon regulator factor 3
LPS: Lipopolysaccharide
Luc: Luminescence
MCP-1: Monocyte chemoattractant protein-1
miRNAs: MicroRNAs
NF-κB: Nuclear factor kappa B
PAMPs: Pathogen-associated molecule patterns
PBS: Phosphate-buffered saline
PPP2R2A: Protein phosphatase 2 regulatory subunit B alpha
PRRs: PATTERN recognition receptors
PTGS2: Prostaglandin-endoperoxide synthase 2
RIG-I: Retinoic acid inducible gene-I
RT-PCR: Reverse-transcription polymerase chain reaction
SD: Standard deviation
siRNA: small-interfering RNA
TFs: Transcription factors
TLR3: Toll-like receptor 3
TLR4: Toll-like receptor 4
TNF: Tumor necrosis factor
TRITC: Tetramethylrhodamine
UTR: Untranslated region
WT: Wild-type
