# Supplementary figures and images for: CircRNA-9119 suppresses poly I:C induced inflammation in Leydig and Sertoli cells via TLR3 and RIG-I signal pathways
Source: Mol Med. 2019 Jun 13;25:28. doi: 10.1186/s10020-019-0094-1 (PMC6567632; doi:10.1186/s10020-019-0094-1)

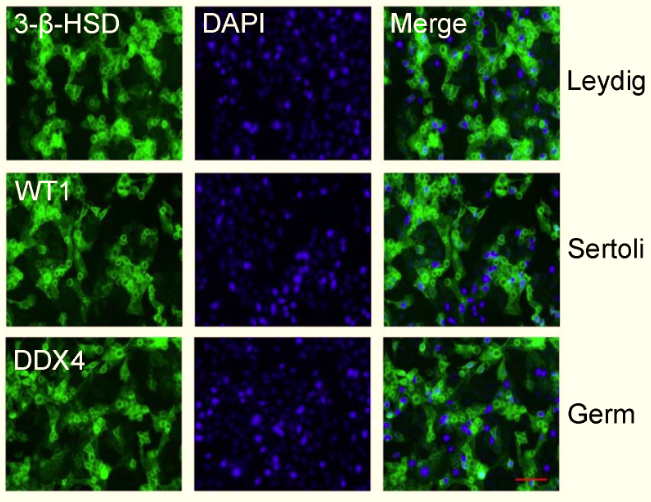


Additional file 1: Figure S1 Immunofluorescence assay of Leydig, Sertoli, and Germ cells.

Supplement: Supplementary file 1 — Figure S1. Immunofluorescence assay of Leydig, Sertoli, and Germ cells. (DOCX 469 kb) [file 10020_2019_94_MOESM1_ESM.docx]
